# Low-Dose Tocilizumab With High-Dose Corticosteroids in Patients Hospitalized for COVID-19 Hypoxic Respiratory Failure Improves Mortality Without Increased Infection Risk

**DOI:** 10.1177/10600280211028882

**Published:** 2021-06-28

**Authors:** Shari B. Brosnahan, Xian Jie Cindy Chen, Juri Chung, Diana Altshuler, Shahidul Islam, Sarun V. Thomas, Megan D. Winner, Allison A. Greco, Jasmin Divers, Peter Spiegler, Daniel H. Sterman, Sam Parnia

**Affiliations:** 1 New York University Langone Health, NY, USA; 2 New York University Langone Hospital – Long Island, NY, USA

**Keywords:** corticosteroids, adult respiratory distress syndrome, respiratory infections, interleukins, medication therapy management

## Abstract

**Background:**

Severe hypoxic respiratory failure from COVID-19 pneumonia carries a high mortality risk. There is uncertainty surrounding which patients benefit from corticosteroids in combination with tocilizumab and the dosage and timing of these agents. The balance of controlling inflammation without increasing the risk of secondary infection is difficult. At present, dexamethasone 6 mg is the standard of care in COVID-19 hypoxia; whether this is the ideal choice of steroid or dosage remains to be proven.

**Objectives:**

The primary objective was to assess the impact on mortality of tocilizumab only, corticosteroids only, and combination therapy in patients with COVID-19 respiratory failure.

**Methods:**

A multihospital, retrospective study of adult patients with severe respiratory failure from COVID-19 who received supportive therapy, corticosteroids, tocilizumab, or combination therapy were assessed for 28-day mortality, biomarker improvement, and relative risk of infection. Propensity-matched analysis was performed between corticosteroid alone and combination therapies to further assess mortality benefit.

**Results:**

The steroid-only, tocilizumab-only, and combination groups showed hazard reduction in mortality at 28 days when compared with supportive therapy. In a propensity-matched analysis, the combination group (daily equivalent dexamethasone 10 mg and tocilizumab 400 mg) had an improved 28-day mortality compared with the steroid-only group (daily equivalent dexamethasone 10 mg; hazard ratio (95% CI) = 0.56 (0.38-0.84), *P* = 0.005] without increasing the risk of infection.

**Conclusion and Relevance:**

Combination of tocilizumab and corticosteroids was associated with improved 28-day survival when compared with corticosteroids alone. Modification of steroid dosing strategy as well as steroid type may further optimize therapeutic effect of the COVID-19 treatment.

## Introduction

Mitigation of inflammation improves survival in patients with severe acute respiratory syndrome coronavirus 2 (SARS-CoV-2) infection resulting in COVID-19 and hypoxemia.^1^ Although initially there was hesitancy to use corticosteroids given harm demonstrated in prior outbreaks,^2^ the randomized evaluation of COVID-19 therapy (RECOVERY) trial demonstrated mortality benefit in COVID-19 patients who required supplemental oxygen.^3^ Given the rapid evolution of standard of care, other studies evaluating the efficacy and safety of corticosteroids in COVID-19 patients were halted.^4-6^ These halted studies left unanswered questions surrounding the most efficacious dose and formulation of corticosteroids.

Nonsteroid immunomodulating pharmaceuticals have been investigated in the treatment of COVID-19 because elevation in inflammatory cytokines, including interleukin (IL)-6 and tumor necrosis factor α, was correlated with viral replication, severity of illness, and outcome, including mortality.^7,8^ Tocilizumab, a recombinant humanized anti-human IL-6 receptor monoclonal antibody, has been shown to have a role in the treatment of COVID-19; however, the optimal timing and dosage are unknown.^9-11^

The combination of tocilizumab and steroids has been explored in several studies. A prospective, historical control study demonstrated that combination therapy had a higher probability of improvement in the World Health Organization 7-point ordinal scale, less progression to invasive mechanical ventilation, and a lower mortality.^12^ The REMAP-CAP study showed a mortality benefit when patients were given dexamethasone 6 mg per day followed by tocilizumab or sarilumab over dexamethasone alone in patients with elevated inflammatory markers who were admitted to their intensive care unit (ICU).^13^ A similar benefit was also noted in the RECOVERY trial with tocilizumab.^14^ Our study seeks to confirm the mortality benefit of combination therapy, characterize biomarker reaction, and chronicle clinical secondary infection to help guide the optimal dose of steroids and tocilizumab in combination for severely hypoxic COVID-19 patients.^15,16^

## Materials and Methods

### Study Design

We performed a retrospective, multihospital cohort study of patients admitted from March 1 to June 25, 2020, to the New York University (NYU) Langone Health System to evaluate the efficacy of corticosteroids in conjunction with tocilizumab in COVID-19 patients with severe hypoxic respiratory failure. The NYU Grossman School of Medicine Institutional Review Board approved the study.

Patients were included if they were admitted to any of the 4 hospitals within the health system, were ≥18 years old, had a positive SARS-CoV-2 polymerase chain reaction (PCR) test by nasopharyngeal swab, had a fraction of inspired oxygen (FiO_2_) ≥70% at any time during their hospitalization, and did not die within 24 hours of the index date. The index date was defined as the first date of medication given when the FiO_2_ was ≥70%. In the combination group, the index date was the first date of administration of the second medication, and in the control group, this was the first date the patient’s FiO_2_ was ≥70%. Patients were excluded if they started a medication 96 hours before FiO_2_ was ≥70% or more than 30 days after (1) testing positive for SARS-CoV-2 or (2) requiring FiO_2_ ≥70%. The control group comprised patients who were SARS-CoV-2 positive with FiO_2_ ≥70% and who received neither tocilizumab nor corticosteroids. The use of FiO_2_ ≥70% was used over patient location or PaO_2_/FiO_2_ ratio in nonintubated patients because these are both dependent on other factors (such as hospital policy, bed availability, and minute ventilation) not intrinsic to the degree of hypoxia.^16^ All patients at the time received other concomitant COVID-19 treatments.

### Study Variables

Data were extracted from the NYU Langone Health COVID-19 clinical database and manual chart review. Observations between 96 hours before and 14 days after the index date were included. Demographic variables included age, body mass index (BMI), sex, race, smoking/vaping status, and Elixhauser comorbidities.^17^ Laboratory variables included were ferritin, C-reactive protein (CRP), D-dimer, lactate dehydrogenase (LDH), troponin, white blood cell count, procalcitonin, IL-6, blood culture, Fungitell, T2Candida, cytomegalovirus (CMV) viral load, and arterial partial pressure of oxygen. Concomitant medication use included COVID-19 therapy (antivirals, ascorbic acid, zinc, famotidine, immune globulins, hydroxychloroquine), vasopressors, anticoagulation, antiplatelets, alteplase, angiotensin-converting enzyme (ACE) inhibitors or angiotensin receptor blockers (ARBs), and paralytics. Clinical data included oxygen support and the modified Sequential Organ Failure Assessment (SOFA) score.

### Outcomes

Our primary outcome was 28-day mortality. If a patient was discharged from the hospital before 28 days, they were censored to survive. Our secondary outcome was a 14-day change in inflammatory biomarkers and the rate of clinically apparent secondary infections. Secondary infections were defined as a positive culture result in a clinically ordered sample.

### Statistical Analysis

Demographic characteristics were categorized by group and presented using the median (interquartile range [IQR]) or frequency (percentage). Continuous variables were assessed for normality using the Kolmogorov-Smirnov test, histogram, and Q-Q plot. The baseline characteristics were compared between groups using the Kruskal-Wallis or Wilcoxon rank-sum test for continuous variables and the χ^2^ or Fisher exact test for categorical variables.

The primary endpoint of 28-day mortality was compared via the Kaplan-Meier method. The log-rank test was used to compare the survival curves between the treatment groups. Sidak adjustment method was used to correct for multiple comparisons. Time to death was compared between treatment groups using the extended Cox regression model because the proportional hazard assumption did not hold. The final multivariable model was determined using the stepwise selection method from an exhaustive list of variables (Table 1). We first performed a series of univariate Cox proportional hazard regression models for the “time to 28-day mortality” outcome using each variable in Table 1 and a list of concomitant medications (vasopressors, zinc, ACE/ARB, famotidine, intravenous immunoglobulin, ascorbic acid, treatment anticoagulation, prophylaxis anticoagulation, direct-acting oral anticoagulants, antiplatelet, alteplase and paralytic for acute respiratory distress syndrome) as a covariate. We then entered all variables with univariate *P* value <0.25 (35 variables) into the stepwise model. We specified 0.15 and 0.10 as variable entry and removal significance thresholds in the stepwise model. This process revealed the final selected model that includes 12 variables with *P* values <0.05.

**Table 1.** Demographic and Clinical Characteristics of Patients in All Groups.

	Steroid only (n = 314)	Tocilizumab only (n = 125)	Steroid + Tocilizumab (n = 223)	Controls (n = 505)	*P* value^a^
Demographics					
Age (years)	68.1 (59.7-76.7)	59.2 (48.2-69.3)	63.0 (55.4-70.7)	69.5 (59.5-81.4)	<0.001
BMI (kg/m^2^)	27.5 (24.3-32.3)	28.6 (24.9-34.3)	29.1 (25.5-33.1)	27.4 (24.2-32.6)	0.059
Female gender	111 (35.4%)	35 (28.0%)	60 (26.9%)	165 (32.7%)	0.151
Race/Ethnicity					0.169
White	124 (39.5%)	55 (44.0%)	93 (41.7%)	215 (42.6%)	
Black	35 (11.2%)	11 (8.8%)	34 (15.3%)	55 (10.9%)	
Hispanic	68 (21.7%)	20 (16.0%)	26 (11.7%)	96 (19.0%)	
Asian/Pacific Islander	32 (10.2%)	15 (12.0%)	18 (8.1%)	43 (8.5%)	
Other/Unknown	55 (17.5%)	24 (19.2%)	52 (23.3%)	96 (19.0%)	
Current/Former smoker	84 (32.2%)	30 (27.8%)	54 (28.9%)	121 (28.8%)	0.758
Current/Former vaping	10 (4.0%)	3 (2.9%)	7 (4.0%)	13 (3.2%)	0.907
Elixhauser comorbidities					
Alcohol use	20 (6.4%)	1 (0.8%)	4 (1.8%)	18 (3.6%)	0.011
HTN with complication	118 (37.6%)	19 (15.2%)	60 (26.9%)	207 (41.0%)	<0.001
HTN without complication	234 (74.5%)	73 (58.4%)	160 (71.7%)	381 (75.4%)	0.002
DM with complication	126 (40.1%)	29 (23.2%)	76 (34.1%)	177 (35.0%)	0.01
DM without complication	136 (43.3%)	49 (39.2%)	93 (41.7%)	230 (45.5%)	0.553
CHF	66 (21.0%)	13 (10.4%)	18 (8.1%)	132 (26.1%)	<0.001
Chronic pulmonary disease	89 (28.3%)	18 (14.4%)	54 (24.2%)	121 (24.0%)	0.023
Renal failure	114 (36.3%)	19 (15.2%)	55 (24.7%)	182 (36.0%)	<0.001
AIDS	2 (0.6%)	0 (0.0%)	3 (1.3%)	4 (0.8%)	0.723
Metastatic cancer	14 (4.5%)	2 (1.6%)	6 (2.7%)	21 (4.2%)	0.392
Solid tumor without metastasis	44 (14.0%)	13 (10.4%)	22 (9.9%)	65 (12.9%)	0.45
Lymphoma	8 (2.5%)	3 (2.4%)	3 (1.3%)	9 (1.8%)	0.739
Liver disease	52 (16.6%)	14 (11.2%)	38 (17.0%)	56 (11.1%)	0.049
Blood loss anemia	12 (3.8%)	1 (0.8%)	8 (3.6%)	10 (2.0%)	0.18
Cardiac arrhythmias	183 (58.3%)	43 (34.4%)	109 (48.9%)	233 (46.1%)	<0.001
Coagulopathy	143 (45.5%)	39 (31.2%)	100 (44.8%)	173 (34.3%)	<0.001
Deficiency anemia	43 (13.7%)	8 (6.4%)	14 (6.3%)	60 (11.9%)	0.014
Depression	67 (21.3%)	29 (23.2%)	45 (20.2%)	97 (19.2%)	0.748
Drug abuse	12 (3.8%)	2 (1.6%)	3 (1.3%)	12 (2.4%)	0.325
Fluid and electrolyte disorders	290 (92.4%)	91 (72.8%)	201 (90.1%)	435 (86.1%)	<0.001
Hypothyroidism	56 (17.8%)	21 (16.8%)	28 (12.6%)	70 (13.9%)	0.278
Other neurological disorders	124 (39.5%)	26 (20.8%)	61 (27.4%)	173 (34.3%)	<0.001
Paralysis	15 (4.8%)	3 (2.4%)	5 (2.2%)	28 (5.5%)	0.157
Pulmonary circulation disorders	51 (16.2%)	21 (16.8%)	40 (17.9%)	85 (16.8%)	0.966
Psychoses	7 (2.2%)	2 (1.6%)	2 (0.9%)	12 (2.4%)	0.609
Peptic ulcer disease	17 (5.4%)	3 (2.4%)	5 (2.2%)	13 (2.6%)	0.13
Peripheral vascular disorders	61 (19.4%)	14 (11.2%)	17 (7.6%)	91 (18.0%)	<0.001
Rheumatoid arthritis	19 (6.1%)	6 (4.8%)	11 (4.9%)	10 (2.0%)	0.022
Valvular disease	36 (11.5%)	8 (6.4%)	16 (7.2%)	61 (12.1%)	0.087
Weight loss	82 (26.1%)	25 (20.0%)	65 (29.1%)	96 (19.0%)	0.009
Weighted Elixhauser score	20 (13-30)	11 (3-23)	18 (10-26)	18 (9-28)	<0.001
Disease severity					
SOFA score	8 (3-10)	1 (0-4)	7 (7-9)	3 (1-7)	<0.001
ICU admission	251 (79.9%)	50 (40.0%)	185 (83.0%)	214 (42.4%)	<0.001
FiO_2_ (%)	100 (100-100)	100 (100-100)	100 (100-100)	100 (100-100)	0.589
Intubation	230 (73.2%)	45 (36.0%)	166 (74.4%)	212 (42.0%)	<0.001
Mechanical ventilator	241 (76.8%)	54 (43.2%)	169 (75.8%)	230 (45.5%)	<0.001

Abbreviations: BMI, body mass index; FiO_2_, fraction of inspired oxygen; HTN, hypertension; DM, diabetes mellitus; CHF, congestive heart failure; ICU, intensive care unit; SOFA, Sequential Organ Failure Assessment.

a *P* values are from the Kruskal-Wallis test for continuous variables and the χ^2^ or Fisher exact test for categorical variables. Continuous data are presented as median (interquartile range).

The propensity score matching (PSM) technique using a greedy matching algorithm balanced the groups. Age, race, sex, BMI, all Elixhauser comorbidities, invasive mechanical ventilation, treatment dose anticoagulation, and prophylactic dose anticoagulation were used as covariates in the PSM model. The Cox proportional hazard model stratified on matched pairs was used to compare the time from hospitalization to death. The χ^2^ test was used to assess the rate of infections between the matched pairs.

A mixed-effects model for repeated measures was used to assess treatment effects on inflammatory biomarkers (ferritin, D-dimer, CRP, and LDH) using baseline values for respective biomarkers, group, time, and the 2-way interaction term between group and time as covariates. All the biomarkers were log-transformed because they had right-skewed distributions. Biomarker data were depicted using percentage change from the baseline on a log scale. SAS 9.4 and R 3.6.3 were used to perform all analyses.

## Results

### Enrollment and Baseline Demographics

Between March 1 and June 25, 2020, a total of 4459 patients were admitted with a positive SARS-CoV-2 PCR test. Out of the 4459 patients, 1332 patients had a FiO_2_ of ≥70% at any time, and 1167 met the other inclusion criteria. Patients were divided into 4 groups: (1) 314 patients received corticosteroids only; (2) 125 patients received tocilizumab only; (3) 223 patients received both corticosteroids and tocilizumab (combination group), and (4) 505 patients did not receive corticosteroids or tocilizumab (control group; Figure S1, available online).

The gender distribution between all groups was similar (*P* = 0.151). The median BMI for the groups ranged from 27.4 to 29.1 kg/m^2^ (*P* = 0.059). The median age for the groups ranged between 59.2 and 69.5 years (*P* < 0.001). The corticosteroid and combination groups had higher median (IQR) weighted Elixhauser comorbidity scores [20 (13-30) and 18 (10-26), respectively] and SOFA scores [8 (3-10) and 7 (7-9), respectively) compared with the other groups. The corticosteroid and combination groups were significantly more ill because a higher percentage of patients in these 2 groups received vasopressors, anticoagulation, and paralytics for ARDS and were intubated when compared with the tocilizumab-only and control groups (Table 1).

### Medication Dosing

The formulation and dose of corticosteroids reported in our study varied based on physician discretion. Corticosteroid formulations given included methylprednisolone, dexamethasone, hydrocortisone, and prednisone. The average daily dose of steroids in both the steroid-only and combination groups was 10 mg dexamethasone equivalent with treatment durations of 6 and 8 days, respectively (Table 2). The majority of our patients received methylprednisolone followed by dexamethasone in both steroid and combination groups. A total of 90% of patients in the combination group and 98% of patients in the tocilizumab group received 400 mg of tocilizumab as a single dose. Tocilizumab was given before corticosteroids 83% of the time; most patients received tocilizumab within 3 days before steroid administration.

**Table 2.** Corticosteroid Type and Dose.

Type of steroid	Steroid-only group				Combination group				*P* value
	n (%)	Average daily dose (mg)	Average duration (days)	Dexamethasone equivalent (mg)	n (%)	Average daily dose (mg)	Average duration (days)	Dexamethasone equivalent (mg)	
Methylprednisolone	213 (67.83%)	94.71	5.40	17.8	181 (81.17%)	85.39	7.18	16	
Dexamethasone	40 (12.74%)	12.19	4.89	12	18 (8.07%)	13.77	8.3	13.77	
Hydrocortisone	38 (12.1%)	172.26	5.72	6.46	10 (4.48%)	170.87	5.98	6	
Prednisone	23 (7.3%)	28.77	7.27	4.32	14 (6.28%)	23.93	11.61	3.59	
Average			5.61	10.15			7.73	9.84	<0.001

### Outcomes

**Figure 1.**
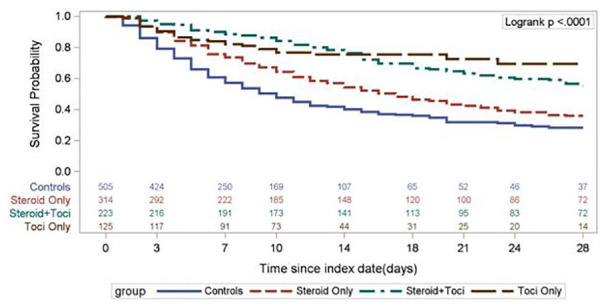
28-Day mortality across all groups. Abbreviation: Toci, tocilizumab.

The crude 28-day hospital mortality was 56% for the steroid only, 24% for the tocilizumab only, 36% for the combination therapy, and 56% for the control group. In unadjusted Kaplan-Meier analysis, groups receiving tocilizumab only or in combination with corticosteroids demonstrated an improvement in survival over the steroid-only and control groups (overall log-rank *P* < 0.0001; Figure 1). Multiple pairwise comparisons showed no difference in survival between the corticosteroid-only and tocilizumab-only groups (Sidak adjusted *P* = 0.049). In time-dependent Cox regression, all treatment groups saw independent benefits with respect to survival over the control group.

When adjusted for covariates (including age, SOFA score, concomitant medication use [prophylactic and treatment anticoagulants, famotidine, ascorbic acid, ACE inhibitor/ARB], and comorbidities [lymphoma, depression, weight loss]), hazard ratios (95% CIs) at day 10 of follow-up were as follows (Table S1, available online): corticosteroid only 0.75 (0.56-1.01), tocilizumab only 0.51 (0.29-0.88), and combination therapy 0.64 (0.46-0.89).

### Matched Propensity Analysis

**Figure 2.**
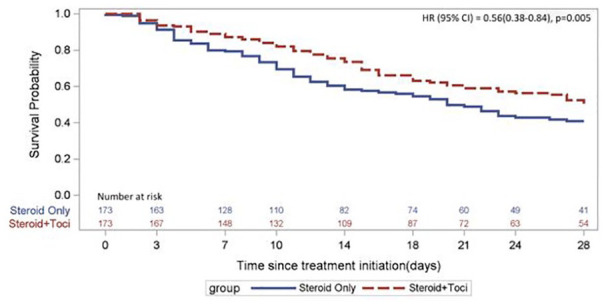
28-Day mortality for the propensity-matched group. Abbreviations: HR, hazard ratio; Toci, tocilizumab.

We performed a propensity-matched analysis for our primary end point—time to mortality—comparing patients in the combination group with patients receiving corticosteroids alone. Among 346 matched patients (173 patients in each group) with well-balanced baseline characteristics (Table S2), the group receiving combination therapy had a 44% reduced hazard of death at 28 days compared with the hazard ratio for the steroid-only group: hazard ratio (95% CI) = 0.56 (0.38-0.84); *P* = 0.005 (Figure 2).

### Longitudinal Biomarkers

We examined the change in biomarkers (LDH, CRP, D-dimer, and ferritin) over time in all groups using mixed-effects models for repeated measurements and log-transformed values. At 14 days, both tocilizumab [estimate (SE) = −0.066 (0.01); *P* < 0.0001] and the combination group [−0.05 (0.007); *P* < 0.0001] had a greater reduction in D-dimer than the corticosteroids-only group [−0.014 (0.007); *P* = 0.032] compared with controls (Table S3). The combination group did not have a significant reduction in ferritin compared with the control group (*P* = 0.466). Tocilizumab alone had a significant reduction in LDH [estimate (SE) = −0.013 (0.004); *P* < 0.001), and CRP [estimate (SE) = −0.114 (0.012); *P* < 0.0001)] compared with controls (Table S3). In the propensity-matched sample, the combination group had a significantly greater reduction in D-dimer, CRP, and LDH than the steroid-only group (Table S4).

### Secondary Infections

The rate of clinically recognized secondary infections in the treatment groups was examined. Positive blood cultures were identified in 10.19% in the steroid-only, 4% in the tocilizumab-only, and 13% in the combination group. Median time to positive blood cultures were 9.5 days (1-81) in the steroid-only, 17 days (3-18) in the tocilizumab-only, and 9 days (1-73) in the combination group. CMV viral loads were elevated in 3.5% in the steroid-only and 2.69% in the combination group. None of the patients in the tocilizumab-only group had an elevated CMV viral load. The median time to development of CMV was 36.5 (19-57) days in the steroid-only group and 34.5 (18-76) days in the combination group. Positive Fungitell test occurred 10.51% in the steroid-only group, 0.8% in the tocilizumab-only, and 5.83% in the combination. The median time (IQR) to development of positive Fungitell was 10 days (0-60) in the steroid-only group, 12 days (not applicable) in the tocilizumab-only group, and 17 days (1-40) in the combination group. Clinically ordered positive T2Candida panels occurred in 7.01% in the steroid-only group and in 2.69% in the combination group. None of the patients in the tocilizumab-only group had a positive T2Candida result. The median time to develop a positive T2Candida result was 14 days (2-83) in the steroid-only group and 17 days (1-64) in the combination group.

In the propensity-matched sample, 12.7% in the steroid group versus 11.6% in the combination group (*P* = 0.742) had evidence of positive blood culture. A positive Fungitell test occurred in 10.4% versus 6.9% (*P* = 0.252), positive T2Candida panels occurred in 6.9% vs 6.4% (*P* = 0.829), and elevated CMV viral loads were seen in 4.6% vs 3.5% (*P* = 0.585), respectively. There was no statistically significant difference in the rate of infections between the propensity-matched groups (Table S5).

## Discussion

Combination therapy was superior for 28-day survival compared with corticosteroids alone in both matched and nonmatched analyses. These data support the notion that higher immunosuppression may be beneficial in patients with severe respiratory failure from COVID-19 as well as sequential benefit of tocilizumab over a higher dose of steroid. There was no sign of increased clinically significant secondary infections in the combination therapy.

In the RECOVERY trial, corticosteroids for COVID-19–induced hypoxic respiratory failure have been shown to improve mortality possibly via anti-inflammatory, antifibrotic, and/or vasoconstrictive properties.^3^ However, RECOVERY only examined 1 dosage strategy, and after its publication, all other ongoing studies of steroids were halted.^4-6^ Other studies had examined different steroid formulations, dosing, and durations of treatment, including hydrocortisone 50 mg every 6 hours for 7 days,^4^ dexamethasone 20 mg for 5 days followed by 10 mg for 5 days,^5^ and hydrocortisone continuous IV infusion of 200 mg/d for 7 days followed by a 14-day taper^6^ because there was no clear guidance on which was superior.

The majority of our patients received methylprednisolone, with a median daily dexamethasone equivalent dose of 10 mg, which is nearly double that in the RECOVERY trial. Prior studies of pulmonary and ARDS disease support the use of higher steroid doses. High-dose steroids with at least 20 mg dexamethasone equivalent are needed to achieve a direct effect on cell membrane–associated proteins.^13^ This has been associated with better outcomes in inflammatory pulmonary diseases, such as pneumonitis from autoimmune conditions and lung transplant rejection.^18^ Advertently, ARDS trials also used high-dose steroid of 23 mg dexamethasone equivalent dose with improved mortality.^18-21^

Whereas both the steroid-only and combination groups received the same equivalent dose of dexamethasone 10 mg, the addition of tocilizumab demonstrated mortality benefit over dexamethasone 10 mg alone. It remains unclear if 6 mg of dexamethasone is enough in severe respiratory failure and if there could be a unique benefit of targeted IL-6 blockade over steroids.

Prior to RECOVERY, methylprednisolone was used for the treatment of ARDS because it has good lung penetration related to its pharmacokinetic properties of greater volume of distribution, higher lipid solubility, and higher protein-binding selectivity than prednisolone.^22^ Both dexamethasone and methylprednisolone have similar characteristics of receptor-binding selectivity and protein-binding characteristics. Dexamethasone is a pure glucocorticoid, whereas methylprednisolone has minimal mineralocorticoid activity. Neither dexamethasone nor methylprednisolone binds to transcortin (corticosteroid-binding globulin), whereas both bind to albumin with low affinity and high capacity. This contributes to linear pharmacokinetics, providing a constant amount of concentration to reach glucocorticoid receptors in the body.^23^ There are few direct comparisons of these steroids; however, a small study suggested that a high dose of methylprednisolone may lead to a further reduction in mortality when compared with dexamethasone in COVID-19 patients.^24^ Whereas both can alter the inflammatory pathway at both genomic and through the rapid-onset nongenomic pathways, methylprednisolone has a higher response via the nongenomic pathway compared with dexamethasone, which may be part of the superior response.^24^ Our data further support the use of methylprednisolone providing mortality benefit over supportive therapy, making it a potential alternative for dexamethasone.

Tocilizumab, an IL-6 receptor inhibitor, has been previously studied for its ability to mitigate systemic inflammatory disease states^25,26^ and explored for COVID-19–related inflammation, given the early link between IL-6 and mortality.^7^ COVID-19 cytokine storm is associated with high levels of inflammatory cytokines often IL-6, CRP, and ferritin. Recent studies have demonstrated the benefit of the reduction of biomarkers specifically in association with CRP levels after day 4 of tocilizumab.^9-11^ Our study showed a significant reduction in CRP, ferritin, LDH, and D-dimer levels with tocilizumab.

Studies have demonstrated mixed results regarding mortality and frequency of intubations with the use of tocilizumab.^11,27-29^ However recent trials revealed that when patients admitted with COVID-19 pneumonia not on mechanical ventilation received tocilizumab, it reduced the likelihood of progression to the composite outcome of mechanical ventilation or death with improvement in survival.^13,30^ Although the exact mechanism has not been described, we postulate that tocilizumab-mediated immunosuppression alone may be too limited to provide a long-term benefit in the treatment of COVID-19, but it may have a synergistic role with corticosteroids.

Tocilizumab differs from corticosteroids in its longer half-life of 13 days. REMAP-CAP’s immunomodulatory data from ICU-level patients receiving IL-6 antagonists after corticosteroids had a mortality benefit over those who did not.^13^ We also observed improved mortality with the combination therapy. We used a lower dose of tocilizumab at 400 mg compared with 800 mg, and the majority of our patients received the dose within 72 hours prior to corticosteroids, in stark contrast to the other studies. We believe that pharmacological data support the use of tocilizumab prior to steroids because it ensures dual immunosuppression in the early treatment of COVID disease while helping avoid administration of steroids during the viral phase.

Our patients received a lower dose of tocilizumab with a higher dose of corticosteroids compared with contemporary studies. For tocilizumab, the CORIMUNO trial used 8 mg/kg as the first dose followed by 400 mg on day 3 if clinically necessary.^10^ Other randomized controlled trials used 8 mg/kg, with a maximum dose of 800 mg, with a second dose allowed if clinical improvement was deemed insufficient.^11,13,29,30^ Most of our patients received a one-time dose of 400 mg of tocilizumab, although our population had twice the rate of mechanical ventilation compared with patients in REMAP-CAP who received 800 mg. Our data support alternate dosing of a 1-time 400-mg dose of tocilizumab. This doubles the number of available dosages of tocilizumab and increases the number of patients able to receive the drug. We found that the addition of tocilizumab to corticosteroids did not increase the risk of clinically investigated bacterial, fungal, and CMV infections compared with corticosteroids alone. Our study supports the alternative timing of administration, giving tocilizumab prior to steroid therapy. We call for studies evaluating various tocilizumab and corticosteroid dosing to maximize benefit for individuals and communities.

Limitations of our study include lack of randomization, the retrospective nature, and variable dosing of corticosteroids. Given the rapidly evolving standard of treatment in patients with COVID-19, it is possible that there are more confounders than found in other types of retrospective studies. Our propensity matching was attempted across all 4 groups but limited because of the severity of illness and changing treatment patterns. This limitation is mitigated because the steroid only and combination groups were well matched and able to be compared.

## Conclusion and Relevance

Our retrospective, observational multicenter study demonstrated that the combination of low-dose tocilizumab and moderate-dose corticosteroids has a mortality benefit in severely hypoxic COVID-19 patients when compared with corticosteroids alone. Infection rates did not increase with the addition of tocilizumab compared with steroids alone. Our study supports the use of substitute corticosteroids, such as methylprednisolone, as well as alternate timing of administration with tocilizumab. Further explorations of these options may optimize the therapeutic treatment for COVID-19 patients. We urge other investigators to further explore the benefit of combined immunosuppression and the timing of the administration of the different immunosuppressants, focusing on various dosing strategies and corticosteroid of choice.

## Supplemental Material

sj-JPG-1-aop-10.1177_10600280211028882 – Supplemental material for Low-Dose Tocilizumab With High-Dose Corticosteroids in Patients Hospitalized for COVID-19 Hypoxic Respiratory Failure Improves Mortality Without Increased Infection Risk Click here for additional data file. Supplemental material, sj-JPG-1-aop-10.1177_10600280211028882 for Low-Dose Tocilizumab With High-Dose Corticosteroids in Patients Hospitalized for COVID-19 Hypoxic Respiratory Failure Improves Mortality Without Increased Infection Risk by Shari B. Brosnahan, Xian Jie Cindy Chen, Juri Chung, Diana Altshuler, Shahidul Islam, Sarun V. Thomas, Megan D. Winner, Allison A. Greco, Jasmin Divers, Peter Spiegler, Daniel H. Sterman and Sam Parnia in Annals of Pharmacotherapy

sj-pdf-1-aop-10.1177_10600280211028882 – Supplemental material for Low-Dose Tocilizumab With High-Dose Corticosteroids in Patients Hospitalized for COVID-19 Hypoxic Respiratory Failure Improves Mortality Without Increased Infection Risk Click here for additional data file. Supplemental material, sj-pdf-1-aop-10.1177_10600280211028882 for Low-Dose Tocilizumab With High-Dose Corticosteroids in Patients Hospitalized for COVID-19 Hypoxic Respiratory Failure Improves Mortality Without Increased Infection Risk by Shari B. Brosnahan, Xian Jie Cindy Chen, Juri Chung, Diana Altshuler, Shahidul Islam, Sarun V. Thomas, Megan D. Winner, Allison A. Greco, Jasmin Divers, Peter Spiegler, Daniel H. Sterman and Sam Parnia in Annals of Pharmacotherapy
