# Supplementary figures and images for: Low-Dose Tocilizumab With High-Dose Corticosteroids in Patients Hospitalized for COVID-19 Hypoxic Respiratory Failure Improves Mortality Without Increased Infection Risk
Source: Ann Pharmacother. 2021 Jun 28;56(3):237–44. doi: 10.1177/10600280211028882 (PMC8250585; doi:10.1177/10600280211028882)

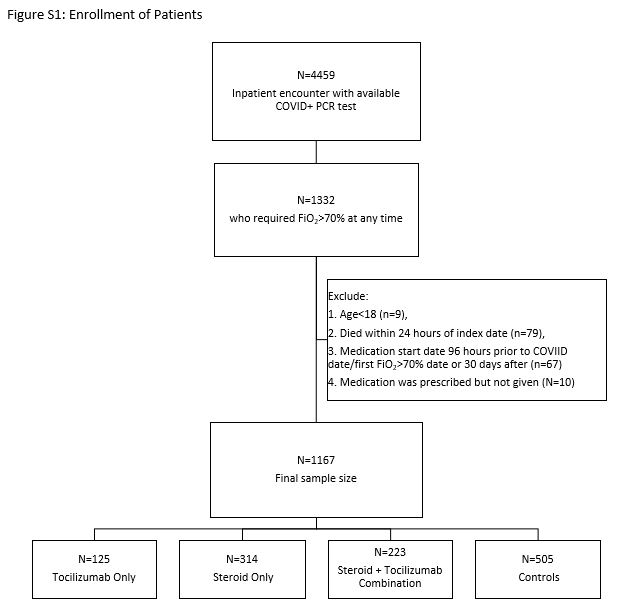

Supplement: sj-JPG-1-aop-10.1177_10600280211028882 – Supplemental material for Low-Dose Tocilizumab With High-Dose Corticosteroids in Patients Hospitalized for COVID-19 Hypoxic Respiratory Failure Improves Mortality Without Increased Infection Risk [file sj-JPG-1-aop-10.1177_10600280211028882.JPG]
