# Supplementary material for: Low-Dose Tocilizumab With High-Dose Corticosteroids in Patients Hospitalized for COVID-19 Hypoxic Respiratory Failure Improves Mortality Without Increased Infection Risk
Source: Ann Pharmacother. 2021 Jun 28;56(3):237–44. doi: 10.1177/10600280211028882 (PMC8250585; doi:10.1177/10600280211028882)
Supplement: sj-pdf-1-aop-10.1177_10600280211028882 – Supplemental material for Low-Dose Tocilizumab With High-Dose Corticosteroids in Patients Hospitalized for COVID-19 Hypoxic Respiratory Failure Improves Mortality Without Increased Infection Risk [file sj-pdf-1-aop-10.1177_10600280211028882.pdf]

Table S1: Time-dependent Hazard Ratios (HR) estimated via Cox regression models for time to death

Treatment group	Time (days)	Time dependent hazard ratios for death: Unadjusted HR (95% CI)	Adjusted HR (95% CI)††
Controls (reference)	---	---	
Steroid	3	0.58(0.45- 0.74)	0.52(0.38- 0.7)
Toci	3	0.34(0.23- 0.5)	0.58(0.36- 0.95)
Steroid+Toci	3	0.19(0.12- 0.3)	0.27(0.17- 0.44)
Steroid	7	0.73(0.6- 0.89)	0.67(0.52- 0.87)
Toci	7	0.31(0.21- 0.46)	0.53(0.32- 0.88)
Steroid+Toci	7	0.35(0.27- 0.46)	0.50(0.36- 0.68)
Steroid	10	0.8(0.63- 1)	0.75(0.56- 1.01)
Toci	10	0.3(0.2- 0.47)	0.51(0.29- 0.88)
Steroid+Toci	10	0.45(0.34- 0.6)	0.64(0.46- 0.89)
Steroid	14	0.87(0.66- 1.15)	0.84(0.59- 1.19)
Toci	14	0.29(0.18- 0.48)	0.49(0.27- 0.9)
Steroid+Toci	14	0.57(0.41- 0.79)	0.82(0.55- 1.2)
Steroid	21	0.97(0.68- 1.38)	0.95(0.62- 1.47)
Toci	21	0.28(0.16- 0.5)	0.47(0.24- 0.92)

Steroid+Toci	21	0.76(0.5- 1.16)	1.09(0.67- 1.79)
Steroid	28	1.05(0.7- 1.58)	1.04(0.63- 1.71)
Toci	28	0.27(0.15- 0.51)	0.46(0.22- 0.95)
Steroid+Toci	28	0.94(0.57- 1.55)	1.35(0.75- 2.41)

†† Adjusted for Age, Sofa Score, Lymphoma, Depression, Weight Loss, ACE/ARB, Famotidine, Ascrobic Acid, Treatment Anticoagulant, Prophylaxis Anticoagulant, and DOACS . Time-dependent HR were estimated via extended Cox regression model.

Table S2: Demographics and Clinical Characteristics Before and After Propensity Score Matching

	Before Match			After Match		
	**Steroid+Toci (n=223)**	**Steroid Only (n=314)**	**P-value[1]**	**Steroid+Toci (N=173)**	**Steroid Only (n=173)**	**P-value[1]**
Demographics						
Age (year)	63.0 (55.4 - 70.7)	68.1 (59.7 - 76.7)	<.001	64.2 (57.1 - 72.3)	65.1 (57.1 - 72.7)	0.330
BMI (kg/m2)	29.2 (25.5 - 33.1)	27.5 (24.3 - 32.3)	0.029	29.1 (25.7 - 32.8)	28.2 (25.3 - 33.9)	0.656
Race/Ethnicity			0.016	53 (30.6%)	54 (31.2%)	0.908
White	93(41.7%)	124(39.5%)				0.967
Black	34(15.3%)	35(11.2%)		68(39.3%)	70(40.5%)	
Hispanic	26(11.7%)	68(21.7%)		26(15.0%)	24(13.9%)	
Asian/Pacific Islander	18(8.1%)	32(10.2%)		25(14.5%)	29(16.8%)	
Other/Unknown	52(23.3%)	55(17.5%)		15(8.7%)	14(8.1%)	
Female gender	60 (26.9%)	111 (35.4%)	0.039	39(22.5%)	36(20.8%)	
Current/Former Smoker	54 (28.9%)	84 (32.2%)	0.455	43 (30.5%)	40 (28.0%)	0.641
Current/Former Vaping	7 (4.0%)	10 (4.0%)	0.989	3 (2.3%)	4 (3.1%)	0.695
Elixhauser comorbidities						
Alcohol use	4 (1.8%)	20 (6.4%)	0.012	4 (2.3%)	1 (0.6%)	0.177
HTN with complication	60 (26.9%)	118 (37.6%)	0.01	52 (30.1%)	50 (28.9%)	0.814
HTN W/O complication	160 (71.7%)	234 (74.5%)	0.474	124 (71.7%)	126 (72.8%)	0.810
DM with complication	76 (34.1%)	126 (40.1%)	0.154	60 (34.7%)	62 (35.8%)	0.822
DM W/O complication	93 (41.7%)	136 (43.3%)	0.711	71 (41.0%)	72 (41.6%)	0.913
CHF	18 (8.1%)	66 (21.0%)	<.001	18 (10.4%)	14 (8.1%)	0.459
Chronic pulmonary disease	54 (24.2%)	89 (28.3%)	0.287	36 (20.8%)	41 (23.7%)	0.519
Renal Failure	55 (24.7%)	114 (36.3%)	0.004	48 (27.7%)	46 (26.6%)	0.809
AIDS	3 (1.3%)	2 (0.6%)	0.4	3 (1.7%)	2 (1.2%)	0.653
Metastatic cancer	6 (2.7%)	14 (4.5%)	0.287	6 (3.5%)	5 (2.9%)	0.760
Solid tumor without metastasis	22 (9.9%)	44 (14.0%)	0.15	19 (11.0%)	15 (8.7%)	0.471
Lymphoma	3 (1.3%)	8 (2.5%)	0.333	3 (1.7%)	5 (2.9%)	0.475
Liver disease	38 (17.0%)	52 (16.6%)	0.883	28 (16.2%)	30 (17.3%)	0.774
Blood loss anemia	8 (3.6%)	12 (3.8%)	0.888	6 (3.5%)	6 (3.5%)	1.000
Cardiac arrhythmias	109 (48.9%)	183 (58.3%)	0.031	87 (50.3%)	92 (53.2%)	0.591

Coagulopathy	100 (44.8%)	143 (45.5%)	0.873	75 (43.4%)	80 (46.2%)	0.589
Deficiency anemia	14 (6.3%)	43 (13.7%)	0.006	13 (7.5%)	10 (5.8%)	0.518
Depression	45 (20.2%)	67 (21.3%)	0.745	29 (16.8%)	30 (17.3%)	0.886
Drug abuse	3 (1.3%)	12 (3.8%)	0.086	3 (1.7%)	3 (1.7%)	1.000
Fluid and electrolyte disorders	201 (90.1%)	290 (92.4%)	0.365	154 (89.0%)	156 (90.2%)	0.725
Hypothyroidism	28 (12.6%)	56 (17.8%)	0.097	23 (13.3%)	27 (15.6%)	0.541
Other neurological disorders	61 (27.4%)	124 (39.5%)	0.004	54 (31.2%)	47 (27.2%)	0.408
Paralysis	5 (2.2%)	15 (4.8%)	0.127	5 (2.9%)	3 (1.7%)	0.475
Pulmonary circulation disorders	40 (17.9%)	51 (16.2%)	0.606	29 (16.8%)	26 (15.0%)	0.660
Psychoses	2 (0.9%)	7 (2.2%)	0.236	2 (1.2%)	2 (1.2%)	1.000
Peptic ulcer disease	5 (2.2%)	17 (5.4%)	0.068	4 (2.3%)	4 (2.3%)	1.000
Peripheral vascular disorders	17 (7.6%)	61 (19.4%)	<.001	17 (9.8%)	15 (8.7%)	0.711
Rheumatoid arthritis	11 (4.9%)	19 (6.1%)	0.579	8 (4.6%)	12 (6.9%)	0.358
Valvular disease	16 (7.2%)	36 (11.5%)	0.098	12 (6.9%)	11 (6.4%)	0.829
Weight loss	65 (29.1%)	82 (26.1%)	0.438	46 (26.6%)	43 (24.9%)	0.713
Weighted Elixhauser score	18 (10 - 26)	20 (13 - 30)	0.019	19 (8 - 27)	18 (11 - 25)	0.852
Disease Severity						
SOFA Score	7 (4 - 9)	8 (3 - 10)	0.067	7 (4 - 9.0)	8 (1 - 10)	0.605
Fio2 (%)	100 (100 - 100)	100 (100 - 100)	0.237	100 (100 - 100)	100 (100 - 100)	0.207
Intubation	166 (74.4%)	230 (73.2%)	0.757	132 (76.3%)	128 (74.0%)	0.619
Mechanical Ventilator	169 (75.8%)	241 (76.8%)	0.795	135 (78.0%)	134 (77.5%)	0.897
ICU admission	185(83.0%)	251(79.9%)	0.377	146(84.4%)	138(79.8%)	0.262
Concomitant Medication						
Treatment Anticoagulation	102 (45.9%)	174 (56.3%)	0.018	84 (48.6%)	88 (50.9%)	0.668
Prophylaxis Anticoagulation	178 (80.2%)	230 (74.4%)	0.122	137 (79.2%)	140 (80.9%)	0.687

[1]P-values are from the Kruskal-Wallis test for continuous variables and Chi-square or Fisher's exact test for categorical variables; Continuous data are presented as median (interquartile range).

Table S3: Changes in biomarkers overtime (≤14 days) in groups

**Outcomes**	**Effects**	**Model Estimate(SE) †**	**P value**
Ferritin (ng/mL)	Time (24 hours)	-0.024(0.003)	<0.0001
	Group*Time		
	Other COVID +	reference	
	Steroid only	0.009(0.004)	0.03
	Toci only	-0.07(0.006)	<0.0001
	Steroid+Toci	0.003(0.004)	0.466
DDimer (ng/mL DDU)	Time (24 hours)	0.005(0.005)	0.321
	Group*Time		
	Other COVID +	reference	
	Steroid only	-0.014(0.007)	0.032
	Toci only	-0.066(0.01)	<0.0001
	Steroid+Toci	-0.05(0.007)	<0.0001
CRP(mg/L)	Time (24 hours)	-0.141(0.007)	<0.0001
	Group*Time		
	Other COVID +	reference	
	Steroid only	0.078(0.009)	<0.0001
	Toci only	-0.114(0.012)	<0.0001
	Steroid+Toci	0.136(0.009)	<0.0001

LDH (U/L)	Time (24 hours)	-0.021(0.002)	<0.0001
	Group*Time		
	Other COVID +	reference	
	Steroid only	0.01(0.003)	<0.001
	Toci only	-0.013(0.004)	<0.001
	Steroid+Toci	-0.001(0.003)	0.713

†Estimated via Mixed-effects Model for Repeated Measures (MMRM) using log-transformed values. The models are adjusted for group and respective baseline values .

Table S4: Changes in biomarkers overtime (≤14 days) in propensity-matched group

		Model	
**Outcomes**	**Effects**	**Estimate(SE) †**	**P value**
Ferritin (ng/mL)	Time (day)	-0.012(0.004)	0.001
	Group (Steroid +Toci vs. Steroid)	-0.194(0.071)	0.006
	Group*Time	-0.009(0.005)	0.078
	Baseline Ferritin	0.671(0.034)	<0.0001
DDimer (ng/mL DDU)	Time (day)	0.001(0.005)	0.856
	Group (Steroid +Toci vs. Steroid)	0.193(0.095)	0.042
	Group*Time	-0.035(0.007)	<0.0001
	Baseline Ddimer	0.450(0.039)	<0.0001
CRP(mg/L)	Time (day)	-0.069(0.008)	<0.0001
	Group (Steroid +Toci vs. Steroid)	-0.959(0.126)	<0.0001
	Group*Time	0.065(0.01)	<0.0001
	Baseline CRP	0.533(0.052)	<0.0001
LDH (U/L)	Time (day)	-0.012(0.002)	<0.0001
	Group (Steroid +Toci vs. Steroid)	0.079(0.042)	0.062

Group*Time	-0.01(0.003)	0.001
Baseline LDH	0.580(0.048)	<0.0001

Table S5: Rate of Infections between Steroid Only and Combination Group

	Full Sample			Propensity Matched Sample		
**Variable**	**Steroid (N=314)**	**Steroid+Toci (N=223)**	**P-value†**	**Steroid (N=173)**	**Steroid+Toci (N=173)**	**P-value†**
Positive Blood culture	30(9.6%)	27(12.1%)	0.344	22(12.7%)	20(11.6%)	0.742
Fungitell	32(10.2%)	13(5.8%)	0.072	18(10.4%)	12(6.9%)	0.252
CMV	11(3.5%)	6(2.7%)	0.596	8(4.6%)	6(3.5%)	0.585
T2 Candida	20(6.4%)	13(5.8%)	0.797	12(6.9%)	11(6.4%)	0.829

† P-values are from Chi-Square test
